# Evaluating the feasibility of the Community Score Card and subsequent contraceptive behavior in Kisumu, Kenya

**DOI:** 10.1186/s12889-022-14388-y

**Published:** 2022-10-24

**Authors:** Dickens Otieno Onyango, Katherine Tumlinson, Stephanie Chung, Brooke W. Bullington, Catherine Gakii, Leigh Senderowicz

**Affiliations:** 1 Kisumu County Department of Health, Kisumu, Kenya; 2 Julius Global Health, Julius Centre for Health Sciences and Primary Care, University Medical Centre, Utrecht, Netherlands; 3 Department of Maternal and Child Health, Gillings School of Global Public Health, University of North Carolina at Chapel Hill, Chapel Hill, USA; 4 Carolina Population Center, University of North Carolina at Chapel Hill, Chapel Hill, USA; 5 Department of Epidemiology, Gillings School of Global Public Health, University of North Carolina at Chapel Hill, Chapel Hill, USA; 6 Innovations for Poverty Action-Kenya (IPA-K), Nairobi, Kenya; 7 Department of Obstetrics and Gynecology, School of Medicine and Public Health, University of Wisconsin, Madison, WI USA

**Keywords:** Social accountability, Contraception, Quality of care, Kenya, Mystery clients, Sub-Saharan Africa, Provider bias

## Abstract

**Background:**

Women seeking family planning services from public-sector facilities in low- and middle-income countries sometimes face provider-imposed barriers to care. Social accountability is an approach that could address provider-imposed barriers by empowering communities to hold their service providers to account for service quality. Yet little is known about the feasibility and potential impact of such efforts in the context of contraceptive care. We piloted a social accountability intervention—the Community Score Card (CSC)—in three public healthcare facilities in western Kenya and use a mix of quantitative and qualitative methodologies to describe the feasibility and impact on family planning service provision.

**Methods:**

We implemented and evaluated the CSC in a convenience sample of three public-sector facility-community dyads in Kisumu County, Kenya. Within each dyad, communities met to identify and prioritize needs, develop corresponding indicators, and used a score card to rate the quality of family planning service provision and monitor improvement. To ensure young, unmarried people had a voice in identifying the unique challenges they face, youth working groups (YWG) led all CSC activities. The feasibility and impact of CSC activities were evaluated using mystery client visits, unannounced visits, focus group discussions with YWG members and providers, repeated assessment of score card indicators, and service delivery statistics.

**Results:**

The involvement of community health volunteers and supportive community members – as well as the willingness of some providers to consider changes to their own behaviors—were key score card facilitators. Conversely, community bias against family planning was a barrier to wider participation in score card activities and the intractability of some provider behaviors led to only small shifts in quality improvement. Service statistics did not reveal an increase in the percent of women receiving family planning services.

**Conclusion:**

Successful and impactful implementation of the CSC in the Kenyan context requires intensive community and provider sensitization, and pandemic conditions may have muted the impact on contraceptive uptake in this small pilot effort. Further investigation is needed to understand whether the CSC – or other social accountability efforts – can result in improved contraceptive access.

**Supplementary Information:**

The online version contains supplementary material available at 10.1186/s12889-022-14388-y.

## Background

Family planning protects the health and well-being of women and children, especially in low- and middle-income countries (LMICs) where health and social inequities contribute to high rates of maternal and infant mortality [1]. In Kenya, the government has committed to ensuring universal access to a wide range of high quality and affordable family planning commodities to enable all individuals to achieve their desired family size [2]. Yet nearly 20 percent of Kenyan women give birth before turning 18 years of age and nearly equal numbers have stated a desire to avoid pregnancy but are not using a modern contraceptive method [3]. This early fertility and unmet need for family planning correspond with high maternal and infant mortality in Kenya, which has a maternal mortality ratio of 362 per 100,000 live births and an infant mortality rate of 39 per 1,000 live births [3]. Expanding contraceptive access among Kenyan women with unmet need can not only accelerate efforts to reduce maternal and infant mortality but can also promote reproductive justice in Kenya.

Yet, a growing body of research shows that some healthcare providers in LMICs engage in behaviors that inhibit family planning use, especially among young and unmarried women. Previous studies have documented several provider-imposed barriers experienced by family planning clients which include discrimination based on a client’s age, parity or marital status, demanding partner consent, soliciting informal payment for services, using disrespectful language (i.e., scolding or laughing at clients or treating them with scorn), or rampant absenteeism of providers [4, 5]. In Tanzania and Ghana, family planning providers were found to restrict access to modern methods based on age or parity and required spousal consent [6, 7]. Mystery clients in Western Kenya reported that providers often invoke unnecessary menstrual requirements, demand informal payments, and are frequently absent from work during business hours [4]. These negative provider behaviors pose a major problem for family planning programs, preventing women from using wanted contraceptive methods and contributing to unmet need—yet identifying solutions has been challenging. Prior quality improvement efforts have focused primarily on improving the technical knowledge of providers. Yet, addressing provider-imposed barriers to family planning requires an approach that addresses not only technical competence but also low provider effort and accountability within the communities they serve.

Social accountability is one such approach. Social accountability aims to improve quality of care by empowering communities to hold their service providers accountable for service quality [8–10]. This approach was developed to address challenges such as inadequate supervision and low accountability among service providers in the health and education sectors in LMICs [11, 12]. There is a dearth of rigorous evidence that assesses the feasibility and efficacy of social accountability interventions in addressing provider-imposed barriers to contraceptive uptake. A limited number of experimental studies conducted in Rwanda, Malawi, and Tanzania evaluated the impact of social accountability interventions and reported improvement in service delivery uptake, reduction in under-5 mortality [13], increased client satisfaction, and higher contraceptive use [14, 15]. While promising, social accountability interventions aimed at improving contraceptive uptake in the Kenyan context have yet to be evaluated.

This paper describes a piloted introduction of a specific social accountability intervention—the Community Score Card (CSC)—in three public healthcare facilities in Kisumu County in western Kenya, a region with high unmet need for family planning and where maternal and infant mortality ratios are nearly twice the national rate. Within the constraints of this limited CSC introduction, we seek to assess changes in both actual and perceived family planning service quality and, specifically, the frequency of negative provider behaviors related to informal fees, absenteeism, and provider bias. We also seek to understand the preliminary impact of this intervention as well as any implementation challenges. Therefore, our objectives are to 1.) to assess changes in community perceptions of family planning service delivery following CSC intervention; 2.) to evaluate changes in the frequency of negative provider behavior o (informal payments, provider absenteeism, and method denial) following CSC implementation; 3.) to note changes in the number of family planning patients pre- and post-intervention; and 4.) to document barriers and facilitators to successful CSC implementation in the Kenyan context.

## Methods

We use a variety of data collection methods, including repeated community scorecards, mystery client observations, unannounced visits, focus group discussions, and service statistics. Quantitative data from the community scorecards, mystery client observations, and unannounced visitors were used to assess changes in the frequency or severity of informal payments and absent providers as well as additional barriers identified by community members. Focus group discussions enabled a process evaluation of the barriers and facilitators of the CSC implementation while service statistics were used to assess changes to patient volume following implementation. Taken together, we mix qualitative and quantitative data from these various data sources to evaluate the impact of the CSC on negative provider behaviors and family planning uptake in these three facility-community dyads.

### The intervention: the community score card

The Community Score Card (CSC) was developed by the Cooperative for Assistance and Relief Everywhere, Inc (CARE) in Malawi in 2002 to enable community members and healthcare providers to collectively identify and problem-solve deficiencies in healthcare delivery [14]. The CSC is a tool that allows communities to monitor and evaluate health facility performance. We implemented the CSC in a convenience sample of three public-sector facilities – and their corresponding communities—in Kisumu County, Kenya. Each of the three communities gathered together to 1) identify and prioritize aspects of family planning healthcare delivery in need of improvement, 2) create corresponding indicators, and 3) design and implement a score card to monitor progress towards quality improvement in the specified priority areas. Additionally, service providers at the three target facilities designed and conducted a score card assessment to self-identify areas for improvement as well as highlight necessary resources. Once completed, the patients and providers in each facility-community dyad came together to share findings and negotiate realistic and agreeable solutions to address priority quality deficiencies. Community members and healthcare providers collaboratively developed a concrete action plan and timeline. The action plan was immediately jointly implemented by the providers and community members. Progress towards completion of the action plan was monitored via monthly community meetings held during the next three to four months; at the end of this time, community members scored facility performance a second time to assess whether there was any progress in addressing the priority concerns of the community.

In each community-facility dyad, a group of four to six youth (ages 18 to 30) formed a Youth Working Group (YWG) and led the development and implementation of all CSC activities, as well as the monthly monitoring meetings. The purpose of the YWG was to ensure young, unmarried people had a voice in identifying the specific concerns and challenges they encounter when attempting to access family planning services from public facilities in their community.

### Evaluation activities

We conducted multiple activities to evaluate both CSC feasibility and impact in the Kenyan context. First, assessment with the initial score cards created at the start of the intervention was repeated one time, three to four months after implementation of the action plan. This allowed us to assess community perceptions surrounding service delivery improvements resulting from the CSC intervention. Secondly, to verify community perceptions of family planning service quality, each of the three intervention facilities received a visit from two mystery clients to measure informal payments and respectful treatment as well as one visit from an unannounced enumerator to measure absenteeism and implement a short provider questionnaire. Third, local administrative data documenting the number of women accessing family planning services was reviewed. In addition to these measures, we evaluated the process of implementing the CSC in the Kenyan context using focus group discussions (FGDs) with YWG members as well as with providers at intervention facilities.

### Data collection tools

#### Mystery clients

Mystery clients are trained data collectors who pose as actual clients to observe provider performance. In this evaluation, two mystery clients visited each of the three intervention facilities on different days and each mystery client was assigned a ‘preferred’ method of family planning (one was assigned the injectable and the other the implant). Mystery clients were ages 20–30, were current residents of Kisumu County, and were fluent in the local language. Mystery clients presented themselves as new family planning clients at the intervention facilities at 8:30am. Each mystery client indicated their ‘preferred’ method of family planning in the event the provider asked them which method they preferred to use.

Following the facility visit, the mystery clients immediately documented their observations of the provider’s performance via a short electronic questionnaire that assessed whether the provider solicited an informal payment; any request for payment in this setting is informal given all family planning methods are mandated to be provided free of charge in all public facilities in Kenya. The mystery client questionnaire also assessed provider disrespect or bias against young or unmarried clients by asking whether the mystery client was denied contraception due to age, marital status, or parity or whether they were treated with scorn, scolded, laughed at, or if the provider threatened to withhold contraception. The mystery client questionnaire was structured but also included open text boxes for mystery clients to share unstructured/qualitative observations related to provider bias and quality of care.

#### Unannounced visits

Unannounced visits were conducted by a trained enumerator to assess provider absenteeism and to conduct a short provider questionnaire measuring opinions about the CSC intervention. A roster of family planning providers who offered family planning in the three facilities was obtained at least five days before the unannounced visit with the assistance of health facility managers. A total of 20 healthcare providers (eight male and 12 female) across the three intervention facilities were documented in health facility rosters as regular family planning providers, scheduled to be working on the dates of data collection. On the day of the visit, the enumerators assessed absenteeism based on the roster. The enumerator visited each facility at 9:00am and stayed in the facility until 2:00 pm to assess the availability of staff. Providers were marked as present if they were physically available in the facility at 9:00am (one hour after the official opening time) and absent if they weren’t. For those providers who did not arrive at the facility by 2 pm, the enumerator made contact via phone to complete the short provider questionnaire.

Mystery clients and unannounced visitors collected data from the three intervention facilities in March 2021, approximately one month after the score card was repeated in each participating community and six months after the initial score card meeting. To evaluate changes in family planning service quality, these data were compared with mystery client and unannounced visit data collected with the same instruments and in the same facilities in 2019.

#### Routine family planning service statistics

Routine family planning service statistics for all public-sector facilities in Kisumu were obtained from the Kenya Health Information System. KHIS is a web-based platform that was adapted from the District Health Information System [16]. Aggregate data on the number of clients accessing family planning services, disaggregated by age group (15 to 19, 20 to 24, and 25 and above) is reported by each public facility to KHIS on a monthly basis. Data were obtained for the period of June 2020-May 2021 to allow assessment of service delivery during the four-month intervention period (October 2020-January 2021) as well as the four months preceding and following the intervention period. We calculated the average number of women accessing family planning commodities, over the four-month periods, disaggregated by the above age groups, and compared the numbers in intervention sites to all health facilities of the same level in the subcounty. We also took the total number of women of reproductive age who received family planning each month and divided it by the estimated number of women of reproductive age in the facility’s catchment population to obtain the percentage of women of reproductive age accessing family planning for each month in the intervention sites versus all health facilities of the same level in the subcounty. We then obtained the average for each period by summing the monthly uptake for each four-month period and dividing it by four.

#### Focus group discussions

Focus group discussions (FGDs) were conducted by two trained Kenyan research assistants, one of whom acted as a moderator and the other as a note taker. We conducted a FGD with each of the three YWGs who led the CSC intervention. An additional FGD was conducted with six healthcare providers who were randomly selected from the three facilities participating in CSC activities. While focus groups may contain as many as 12 participants, we intentionally sought smaller groups to encourage more in-depth conversation, a practice endorsed by experts in focus group methodology [17]. FGDs with both YWG members and healthcare providers were designed to document barriers and facilitators to successful CSC implementation. Two researchers (BB, SC) conducted a targeted thematic analysis of focus group data from the three YWGs and one group of providers to assess facilitators of the CSC, barriers to the CSC, successes of the CSC, and deviations from the CSC intervention as originally designed. After a first read of the data, a codebook with both low-level descriptive codes and higher-level specific codes was created for each type of FGD participant: score card leaders and providers. These codebooks were then evaluated by CG, a Nairobi-based research assistant supporting the CSC evaluation. CG checked the consistency of the coding and ensured inclusion of all major themes identified while overseeing all data collection. The data were then coded independently, assessed for inter-coder reliability, and compared.

This study was approved by the University of North Carolina at Chapel Hill ethics review committee and the Kenya Medical Research Institute (KEMRI) scientific and ethical review unit (SERU). Administrative approval was obtained from Kisumu County Department of Health, Kisumu County Department of Education and from County Commissioner Kisumu County. Written informed consent was obtained from all focus group participants and all health care providers who participated in the short provider questionnaire and by all facility managers for the mystery client component. To anonymize participating facilities, we refer to their facility type rather than their facility name. There were no study participants below the age of 18 years. All methods were carried out in accordance with relevant guidelines and regulations.

## Results

### Changes in community perceptions of family planning service quality: results from repeated community score cards

**Fig. 1:**
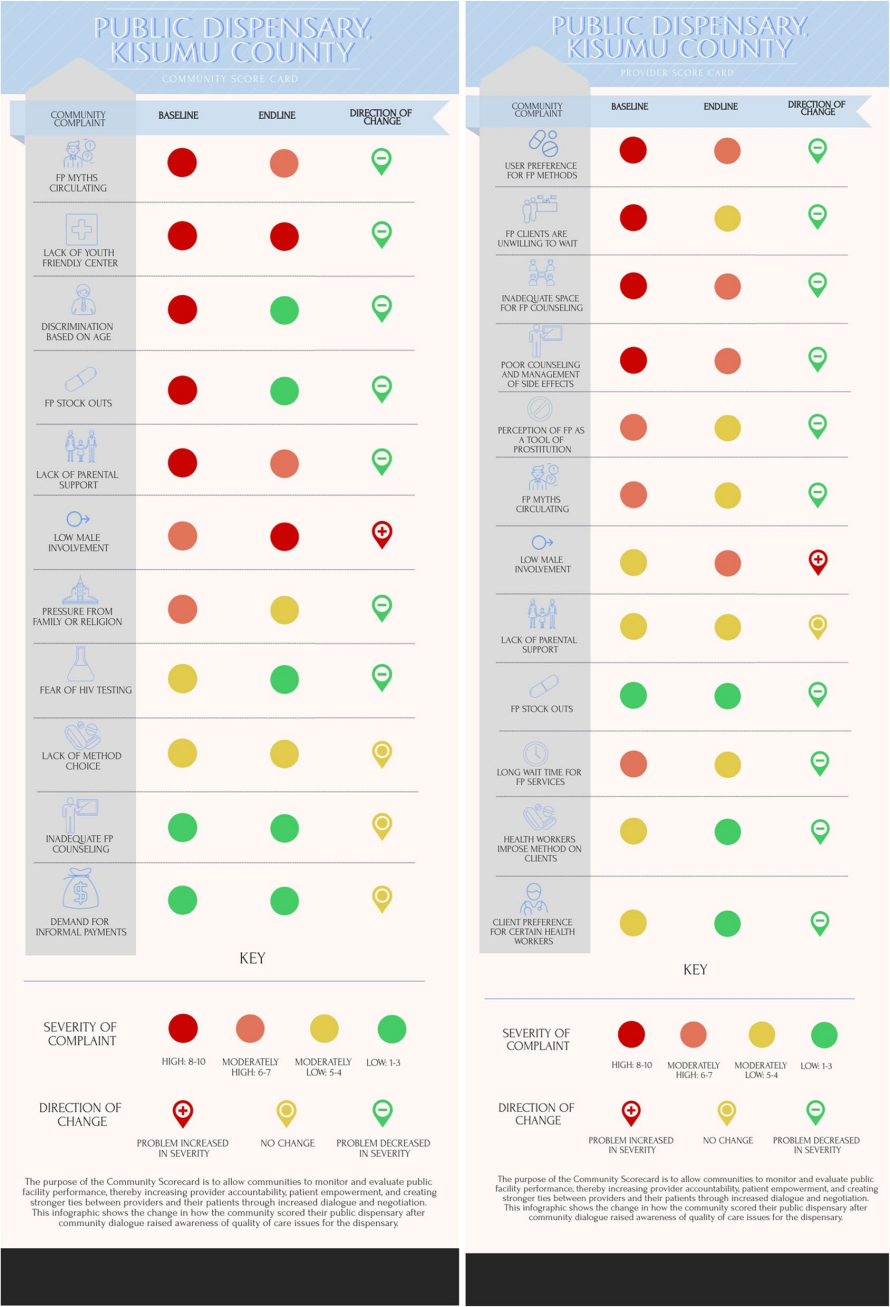
Baseline and endline community score cards developed by community members and health facility staff at the public dispensary in Kisumu County

At the public dispensary, the community identified 11 issues to address (Fig. 1): five of high severity and two each of moderately high severity, moderately low severity, and low severity. Of the high priority issues, all five showed some improvement by the time the score card was repeated, with two (age discrimination and stockouts) showing large improvement. However, the other three high severity issues—the circulation of inaccurate family planning information, lack of a youth friendly center, and parental support for family planning use—remained moderately high severity issues. Of the other six issues, one saw modest improvement, three saw no change (although these were the three lowest priority issues) and low male involvement in family planning use worsened. Interestingly, the providers had also identified low male involvement as one of their issues, and also reported that the severity of the issue had increased. Another area of accord between community and provider was family planning stock-outs: both parties agreed that stock-outs were significantly improved by the repeated score card. Of the 12 issues identified by providers, all four of the high severity issues improved. There was some overlap between the issues identified by community and providers, but half of the issues identified by providers were behavioral changes desired in patients, or gaps in knowledge about family planning that were present in the community.

**Fig. 2:**
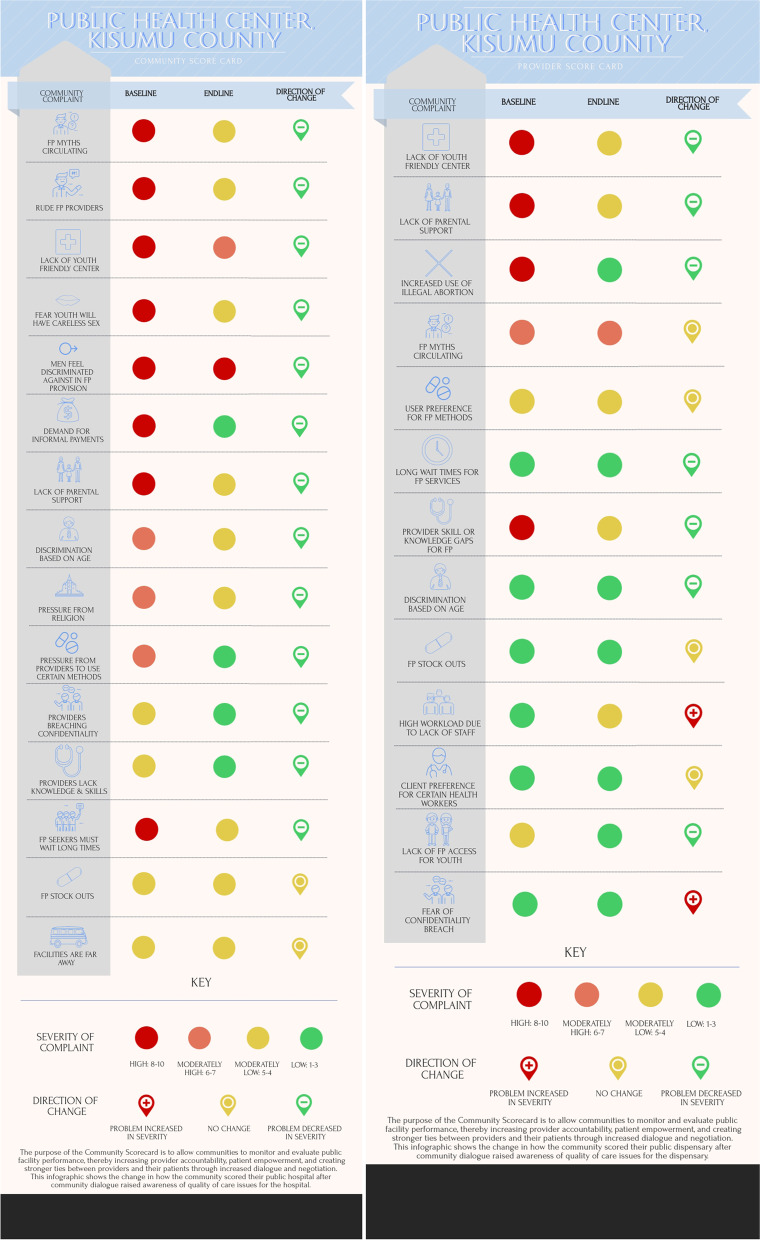
Baseline and endline community score cards developed by community members and health facility staff at the public health center in Kisumu County

The community paired to the public health center reported that eight of their 15 identified issues were of high severity (Fig. 2). Of these, 13 indicators improved after implementation of the action plan, with some indicators improving by a substantial amount, most notably respectful client treatment, informal payments, provider method preferences, and extensive wait times; this suggests substantial improvements to the quality of family planning provision. In contrast with the community-derived score card, only three of the 13 issues identified by providers were seen as high priorities, two of which were also big concerns for the community: lack of a youth friendly center, and poor parental support for youth family planning use. However, the providers did not feel that high priority issues identified by the community, such as long wait times or demand for informal payments were of the same importance for providers. While the public health center community reported that all issues improved or remained the same, the providers did not have similar results across the board. Providers reported that their high workloads had increased in severity and other issues such as stockouts, family planning myths, and user preference for specific methods contraceptives had remained the same. The most drastic improvement seen by providers was a reduction in illegal abortion services, whereas the community saw the most improvement in the rudeness and quality of counseling given by providers.

**Fig. 3:**
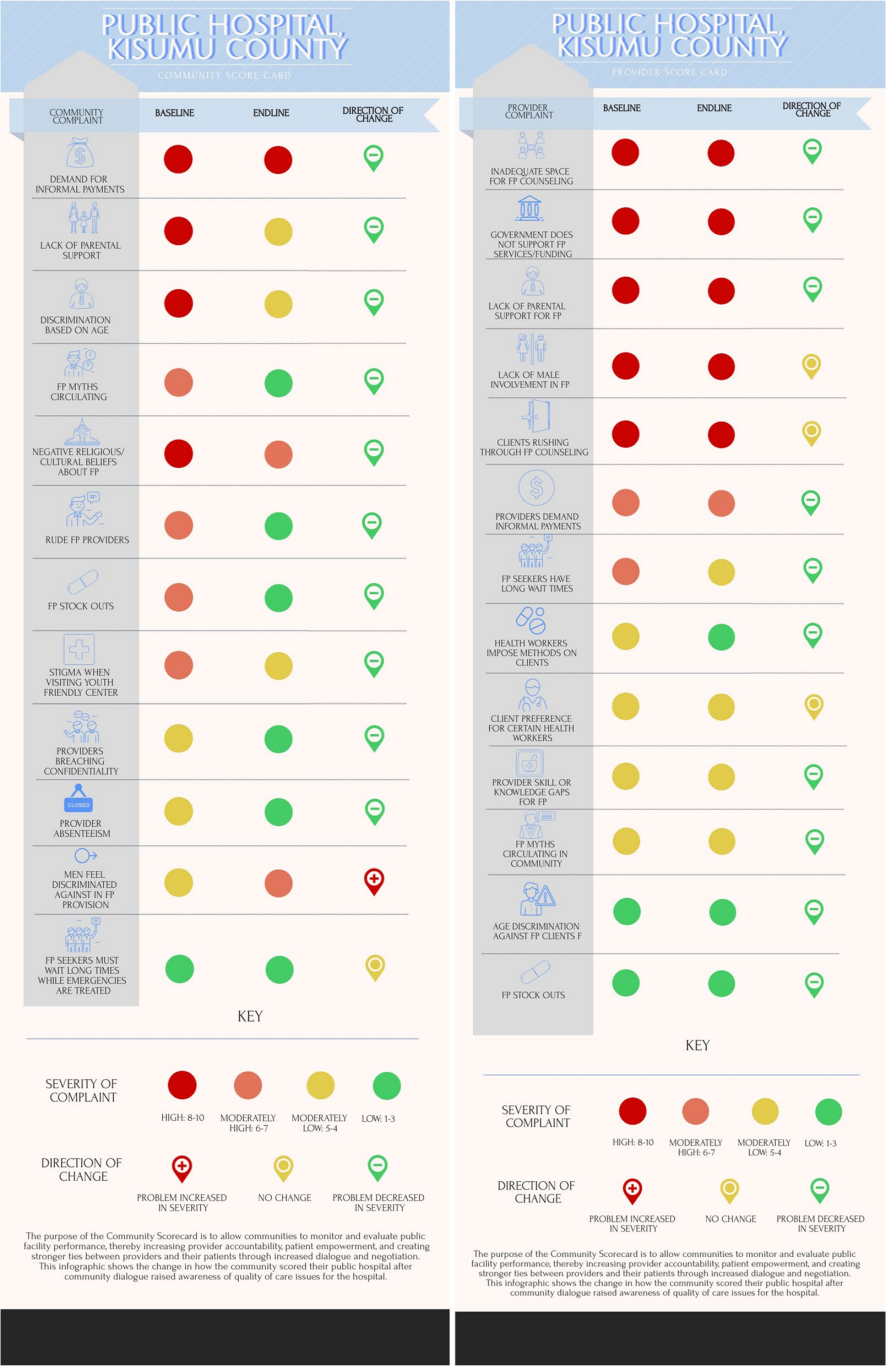
Baseline and endline community score cards developed by community members and health facility staff at the public health center in Kisumu County

The public hospital community identified 12 issues, of which only one increased in severity (Fig. 3). Again, the inclusion of men in family planning was a larger issue than had been seen in the first score card. The public hospital providers also identified male inclusion as an issue but did not see a change in severity. Both the providers and the community agreed that poor parental support for family planning was a significant issue, as was demand for informal payments. Discrimination based on age was also an issue that appeared in both sets of score cards; however, providers ranked this as a low priority issue, and the community ranked this as a high priority issue and did not report the same improvement seen by the providers. Provider absenteeism was an issue identified only by the community, who reported some improvement. Providers were more concerned with the support, funding, and space available in their facility, while the community focused on provider and community behaviors and attitudes.

### Changes in actual service quality: results from mystery clients and unannounced visitors

A comparison of data provided by mystery client and unannounced visits conducted in 2019 (baseline) and again in 2021 (endline) are summarized in Table 1 and described for each of the three participating facilities below.

**Table 1:** Summary of findings from mystery clients^a^ and unannounced visits^b^; Kisumu County, Kenya 2019–2021

	**MCs asked to pay informal fee (%)**	**Instances of provider bias towards young, unmarried, or nulliparous MCs**	**Mean wait time for MCs (minutes)**	**Providers absent during UAVs (%)**
**Dispensary**				
Baseline	NA^c^	1 report (*n* = 3)	62	75% (*n* = 4)
Follow-up	0% (*n* = 2)	0 reports (*n* = 2)	75	100% (*n* = 4)
**Health Center**				
Baseline	33% (*n* = 3)	0 reports (*n* = 3)	69	80% (*n* = 5)
Follow-up	100% (*n* = 2)	1 report (*n* = 2)	6	100% (*n* = 7)
**Hospital**				
Baseline	66% (*n* = 3)	0 reports (*n* = 3)	21	75% (*n* = 6)
Follow-up	50% (*n* = 2)	0 reports (*n* = 2)	64	66% (*n* = 9)

*MC* Mystery client, *UAV* Unannounced visit

^a^Each facility received three MC visits at baseline in 2019 and two MC visits at follow-up in 2021

^b^Each facility received two UAVs (one at 9am and one at 3 pm) at baseline in 2019 and one UAV (at 9am) at follow-up in 2021; the baseline calculation of percent of providers avsent is an average of the two UAVs

^c^Informal fees not measured because all MCs were denied methods due to requirements for HIV and pregnancy tests

#### Pilot facility #1: dispensary

At baseline, all mystery clients were denied a contraceptive method because the facility first required a pregnancy and/or HIV test prior to offering contraception; as such, we were unable to measure informal payments in this facility at baseline. Regarding provider bias, one of the three mystery clients reported she was treated with scorn by the provider, indicating “… *when I assured her (that) I am not pregnant she sneered at me and looked at me badly*.” Despite arriving at the facility by 8:30am, mystery clients reported waiting, on average, just over one hour (62 min) to be seen because providers either arrived late or engaged in ‘storytelling’ until 9:30am. Although there were four providers on the duty roster, three (including the facility manager) were absent at both unannounced visits; the single provider present at both unannounced visits reported that the absent providers were running personal errands at the first unannounced visit and was unsure of the reason for their absence at the second unannounced visit.

Following the CSC intervention, mystery client visits were repeated, with both mystery clients offered family planning methods, all of which appeared to be in stock and offered free of charge – an improvement over baseline. However, a provider did caution one mystery client that the facility charges 200 shillings^1^ for implant removal ‘because it requires a specialist for removal.’ Both mystery clients reported being treated with respect, although one was discouraged from using the intrauterine device because she is nulliparous, with the provider strongly recommending condoms. A similar pattern of wait time was observed at follow-up, with providers arriving at the facility shortly before 9:30am and attending to patients starting around 9:45am, resulting in mystery clients waiting 75 min, on average to be seen. At follow-up, no providers were present when the unannounced visitor arrived at 9am. Two providers arrived late (one at 9:30am, no reason offered, and one at 11:15am, due to running personal errands) while the other two were absent for the whole day, due to authorized personal leave and off-site duties.

#### Pilot facility #2: health center

At baseline, one in three mystery clients was asked to pay an informal fee. One mystery client (implant user) reported that the provider *“said family planning services are free but then I buy him tea at 100 shillings and then he will do the procedure for free*.” No instances of disrespect or of providers refusing to offer family planning for reasons related to age, marriage, or parity were reported at this facility at baseline. However, one mystery client reported their provider discussed future plans to refuse family planning to patients who arrived at the facility without their partner. Two mystery clients waited over an hour to be seen while a third reported she was attended to within 15 min of arriving, for an average wait time of 69 min. There were five providers on the duty roster for this facility; three of these were absent at the first visit by an unannounced enumerator (two were on vacation and another, the manager, was at an off-site meeting) and all providers were absent at the second unannounced visit, as they were running personal errands or were absent for unknown reasons.

After the CSC intervention, both mystery clients were asked to pay an informal fee for family planning, in the amount of 50 shillings, and the provider indicated these fees were sanctioned by the Ministry of Health.^2^ No instances of disrespect were reported and both mystery clients were seen promptly upon arrival (six-minute wait time, on average). At follow-up, our project identified seven providers as routinely offering family planning services and all seven were absent on the day of the unannounced visit at 9:00am, with the first provider arriving at 9:06am. Three of the seven providers were out all day on authorized leave while the other four were late due to running personal errands (with the last three arriving between 9:30 and 10:20am).

#### Pilot facility #3: sub-county hospital

At baseline, informal fees ranging from 50 to 200 Kenyan Shillings and were solicited from two out of three mystery clients, who were attended to within 15 to 45 min of arriving at the facility. Although the baseline wait time was shorter at this facility (on average, 21 min), providers reportedly rushed during family planning counseling, with one mystery client reporting, “*The providers came early but kept on story telling for about 19 min before calling me in. She was also in a hurry hence attended to me hurriedly despite the fact that I was the only one seeking the service*.” No instances of provider disrespect or reluctance to offer family planning to young, unmarried, or nulliparous women were reported. This hospital had six family planning providers on their duty roster; half were absent at the first unannounced visit (primarily due to being on vacation) and all were absent at the second unannounced visit, due to a mix of sanctioned and unsanctioned reasons including being on leave, being on night duty the night before, and being late to work.

At follow-up, one of the mystery clients (implant user) was not offered a method – after waiting over one and a half hours to be seen because the provider was late to work – because implants were stocked out at the time of visit, although the mystery client was informed by her provider that all methods are offered for free at the facility. The other mystery client was attended to by a laboratory technician (untrained provider) who offered her the injectable and then asked her to pay 100 shillings, because ‘*it took an effort*’ to provide her with the service she needed. No instances of provider disrespect or age bias were reported by mystery clients. On average, the two mystery clients waited 64 min to be seen by a provider, a substantial increase over baseline. At follow-up nine providers were routinely offering family planning and three of these were present at the time of the unannounced visit. Of the other six, three arrived late, between 10am and 10:30am. The other three were absent for the entire day, two due to authorized leave and one due to personal errands.

### Changes in family planning patient volume: results from service statistics

Facility-level service statistics are presented in Table 2. Both intervention sites and the average performance in other public facilities of the same level experienced a decrease in the number and proportion of women of reproductive age accessing family planning services. Four months pre-intervention, the proportion of women of reproductive age accessing family planning in the intervention hospital (62%) was higher than the average uptake in all hospitals in the subcounty (55%). The family planning uptake among all women of reproductive age in the intervention hospital reduced by a larger margin than the average of all hospitals in the subcounty. Similarly, the proportion of women of reproductive age accessing family planning services reduced by a larger margin in the intervention health center and dispensary compared to the average of all health centers and dispensaries in the subcounty, respectively. The drop in the number of women accessing family planning was consistent across all age groups. For example, the average number of women aged 15–19 years accessing FP services fell (and by a larger amount in the intervention health center and intervention dispensary compared to the average of facilities of the same level) while there was a modest increase in the number of women aged 20–24 years accessing FP in the intervention hospital and health center.

**Table 2:** Facility-level service statistics for women residing in the facility catchment area per month; Kisumu County, 2020–2021

Time period	Intervention hospital	Mean of all other public hospitals (*n* = 4)	Intervention health center	Mean of all other public health centers (*n* = 9)	Intervention dispensary	Mean of all other public dispensaries (*n* = 21)
	*Percent of all women of reproductive age receiving family planning services each month*					
Monthly average, four months pre-intervention (June-Sept 2020)	62%	55%	64%	57%	64%	45%
Monthly average, four months of intervention (Oct 2020-Jan 2021)	24%	32%	20%	26%	24%	23%
Monthly average, three months post-intervention (Feb-Mar 2021)	29%	38%	40%	45%	33%	40%
	*Number of women 15–19 years of age receiving family planning services each month*					
Monthly average, four months pre-intervention (June-Sept 2020)	40	50	98	42	71	32
Monthly average, four months of intervention (Oct 2020-Jan 2021)	18	17	17	11	6	11
Monthly average, three months post-intervention (Feb-Mar 2021)	40	23	16	12	14	6
	*Number of women 20–24 years of age receiving family planning services each month*					
Monthly average, four months pre-intervention (June-Sept 2020)	107	155	38	40	129	63
Monthly average, four months of intervention (Oct 2020-Jan 2021)	55	73	52	28	52	34
Monthly average, three months post-intervention (Feb-Mar 2021)	125	95	42	43	35	26
	*Number of women 25–29 years of age receiving family planning services each month*					
Monthly average, four months pre-intervention (June-Sept 2020)	37	26	7	4	2	5
Monthly average, four months of intervention (Oct 2020-Jan 2021)	42	20	9	5	1	8
Monthly average, three months post-intervention (Feb-Mar 2021)	25	31	32	16	26	8

It is not possible to present percentages for the figures that are disaggregated by age group because the available data estimating the total number of women of reproductive age in each catchment area (i.e. community) is not disaggregated by age

### Barriers and facilitators of CSC implementation: results from focus group discussions

Table 3 describes the dominant themes that emerged from analysis of FGDs, and provides supportive/illustrative quotes for each theme, which we describe in detail below.

**Table 3:** Emergent themes and illustrative quotes from discussions with three Youth Working Groups and one group of healthcare providers working at intervention facilities; Kisumu County, Kenya 2021

Descriptive Code	Higher-Level Emerging Themes	Illustrative Quote
Facilitators of CSC	YWG: Community health volunteers were essential to the success of the CSC; some communities were eager to participate in the CSC Providers: Willingness of the community to engage in dialogue, openness to change from the providers	*“I got help from a community health volunteer (CHV)… We were walking with the CHV so that she can try and explain that this was just a participation and not giving the family planning. That this is just a participation, a collection of data… So the CHV explained and everything went well.”* -YWG member *“The turn up was good. Mmmh…there are people who were responding full…. But the turn up was good. The link between us and the society was awesome, and they gave us an easy time to communicate with them.”* -YWG member *“I can say that it was an educative experience because we were able to sit with the community representatives and understand how the community views the health facility.”* -Provider
Barriers to CSC	YWG: Stigma around youth use of family planning made parents reluctant to let their children go to CSC meetings; negative provider behaviors persisted after CSC implementation and discouraged participants; communities were wary due to past family planning programs that gave youth family planning without parental permission; covid-19 restrictions Providers: Providers felt frustrated when they felt their actions were justified due to their more advanced medical knowledge; providers felt that youth may not be the right facilitators for the CSC role; providers would like better consideration of their time and more compensation for participating in the CSC	*“There was a time that we were told to bring some youth also so that they can participate in this score card… gathering the youth in my community was such a problem. [When] you approach a …female youth and the parent refuses: “You want to go…you want to take her to family planning without the parents’ consent?” … So even if you try to explain that this is like collection of data the parent won’t agree because sometimes back youths were picked from the community and taken …for family planning without their parents’ consent.”* -YWG member *“What didn’t go well …we were sitting with service providers. When we are seated there, they promise us what they cannot do. It’s like they were just fooling us. So, what they do is different with what they say. And that problem is still present. Like demand for informal payment. We were not able to do anything with that though we tried.”* -YWG member *“You know youths if you pick them to work for a duration, like mine, I know of one who has gone to college, others have moved away, others have gone to Nairobi. So, you find out that it is difficult to work with them… I think this issue should go specifically to community health volunteers. They are always there; they meet these youths everywhere with their parents… so it is even easier managing this thing when we use community health volunteers rather than youths. It is good to use youths, but we try to identify youths who are married. Even if you are married, sometimes the marriage can dissolve, and you go and again that will be a lost asset.”* -Provider
Success of the CSC	YWG: Barriers to family planning use were identified through the scorecard process; youth were proud of their role in the community; some solutions were identified and implemented Providers: There were better relations and open dialogue between providers and community; some providers felt that misunderstandings were cleared up and that they could provide better care to their communities	*“The problems were many in the facility but now when we are going to finish the program, in the last meeting that we had; I heard him saying that most youths are coming to the facility to access the service. Even the ones that were being criticized that were of young age were now coming to get the service.”* -YWG member *“I think some of the things that went well is that the issues that were raised by the community, quite a few of them were implemented. Like that fear of HIV testing. We told them that there is opt out, it is not compulsory, it is optional. And so we advised the health providers that these people fear and so they should give them that opt out.”* -YWG member *“And again, it helped us also to improve our services and also mmmh…helped the community to understand the facility. Like maybe there are some things they did not know about the facility and now they are getting it clearly”* -Provider
Deviations from Original CSC Plan	YWG: Due to stigma around family planning use, youth leaders developed a role of “FP Educator” as they tried to convince people to come to meetings; the CSC was confused for family planning education Providers: Some providers felt that the CSC was a way to teach the community how to be better patients instead of a way of evaluating the clinics	*“My experience was… I learnt a lot of things. I have learnt a lot from the community score card and it has made me educate many about family planning.”* -YWG member *“When it comes to… teaching them maybe how to use condom, the way you do it could make them laugh so much. Okay, they wanted to see it repeatedly more so we just did it because they wanted to know.”* -YWG member *“And after the score card, after we had shared and came up with a way forward, we are able to see now the community come for the services. They have changed their time of coming… They view family planning as a service just like any other, mmmh… they are able to understand, they are able to be patient and wait… At each level, you are able to communicate something, they are able to understand and take it positively. Yeah So, I would say that it is becoming lighter even on our side.”* -Provider

#### Facilitators of the community score card

As seen in Table 3, data from youth focus group discussions indicate that implementation of the CSC was aided by the help of community health volunteers (CHVs). CHVs assisted facilitators in encouraging youth to attend CSC meetings, especially when parents were reluctant or did not trust the youth facilitators. One youth facilitator said that “*We were using the CHVs to help involve the community… It would be difficult to face a parent. Or let’s say it’s a male parent… they don’t believe in the youths. So… we were using the CHVs to help in that area*.” According to youth facilitators and providers, support from community members also aided the score card process. Youth facilitators said community members were “*ready*” (422, 100), “*had willingness to learn more,” “gave us an easy time to communicate with them,”,* and *“were very interactive.”*

Some providers were also open to learning community perspectives and changing their own behaviors, which facilitated the score card process. One provider noted that participating in the score card *“informed [them]. Like maybe we were weak. It was a good forum. Someone could talk what they have at heart.”* When speaking of informal fees charged to community members seeking family planning services, another provider said, *“If it is issues of charging clients and that is making clients not access the services, it should be improved on so that the clients get the services for free.”*

#### Barriers to the community score card

Youth facilitators reported barriers to recruiting youth to attend CSC meetings due to parental reluctance. Parents were sometimes unwilling to allow their children to attend CSC meetings because of stigma against youth using family planning and because past family planning programs recruited youth without parental consent. One facilitator said, “*When you approach a …female youth and the parent refuses: ‘You want to go…you want to take her to family planning without the parents’ consent?’ … So even if you try to explain that this is like collection of data the parent won’t agree because sometimes back youths were picked from the community and taken …for family planning without their parents’ consent.*”

Providers also noted barriers to the extended score card process presented by the mobile and transitory nature of youth. One provider said that, “*You know youths, if you pick them to work for a duration, … one who has gone to college, others have moved away, others have gone to Nairobi. So, you find out that it is difficult to work with them*.” Providers suggested that facilitation by CHVs, rather than youth, could improve the process.

Youth facilitators observed that some negative provider behaviors continued even after providers agreed to address service barriers to family planning. After providers said “*they valued clients in their facility*” during the CSC, negative provider behaviors persisted. In one example, after a community member left a facility without receiving services due to a rude provider, CHVs raised the problem again with the facility. This CHV was told “*the problem would be taken care of. But later on, it’s like they didn’t do anything about it.*” Thus, providers were not only not addressing barriers brought up in the CSC meeting, but also ignoring CHVs who tried to help community members.

Lastly, some providers noted that the score card process was time consuming and noted wanting increased compensation for their time. One provider commented, “*Yes, because you sit for long and you participate, the expectations from the program are high but… we are not appreciated. You know just finding time, sitting down talking, no refreshment, and the reimbursement is little.*”

#### Successes of the community score card

The focus groups with YWG members reported CSC successes included encouraging the community to speak openly about barriers to family planning use, identifying solutions to those barriers, and in some cases, successfully implementing solutions. As one youth facilitator said, “*The experience was good: you get to interact with the youths, you know the problems, the challenges…and what people are going through to access medical services here… and what can be done to help the problems that they are going through.*” The main barriers to family planning usage at the facility level identified by community members included negative provider behaviors, stock-outs, discrimination against adolescents, and informal fees. The CSC process was used to identify several possible solutions, some of which were successful in addressing barriers. For example, the CSC process led to youth being given “*good time*” at health centers, where they were “*advised and talked to about the family planning before they insert it. [Adolescents were] told about the challenges and importance*” of family planning. This led to youth, “*even the ones that were being criticized … now coming to get the service.*” The CSC also identified that provider behaviors, especially speaking harshly to patients, were a significant deterrent. Youth facilitators reported one benefit of the CSC was “*The way they [providers] were talking was just good as compared to the shouting before.*”

From the provider focus group, the main success of the CSC was the increased dialogue and communication between the providers and the community. The providers felt that the CSC “*was an educative experience because we were able to sit with the community representatives and understand how the community views the health facility.*” The providers characterized the meetings as “*so healthy*” and agreed that it was an opportunity for the community to understand the issues facing the clinic, as well as for the healthcare providers to understand the perspective of the community members. Providers also felt that the CSC allowed them to educate the community in ways that improved the healthcare the community members later received, as the community *“[is] able now to access these services with an informed mind.”* Finally, providers were also able to identify areas to improve their practice: for example, one provider said, “*we should also ensure that we provide quality and efficient services to the youth,”* and identified increasing facility staff as a way to increase service quality and efficiency. On a more personal level, providers appreciated the CSC as a way to remind the community that they are only human: “*They are able also to understand that even the health care workers who are working there are also human beings… we try and ensure they understand us as we understand them*.”

#### Deviations from original community score card plan

Unfortunately, both the youth facilitator focus groups and the provider focus groups reported some significant deviations from the original intention of the CSC. The reasons why youth facilitators opted to deviate from their extensive training is unclear. The major deviations revealed by the youth facilitator focus groups centered on two related issues: first, the community misunderstood the role of the score card meetings in the community, believing the meetings were intended as a forum for family planning education rather than a dialogue and action plan for family planning quality improvement. Secondly, the youth CSC facilitators misunderstood their role as CSC facilitators and mistakenly identified more as family planning educators than as CSC facilitators. The combination of these two deviations from the design of the CSC intervention led to several challenges. For example, in some communities, parents were reluctant to let their youth, especially their daughters, attend score card meetings because they believed that these meetings were intended to give their children family planning services or education. This may be in part because so many youth facilitators spoke about educating others about family planning as part of their role as CSC facilitators. Some of this “family planning education” occurred as part of encouraging youth to come to CSC meetings by addressing myths about family planning users, as explained by one facilitator: “*And then we tried to make them now come… like for example they were talking of myths and lack of information. Some people are not…they don’t know anything about the family planning, so talking to them about it made them to change their minds.*” In other cases, it appears that during CSC meetings, youth were asking questions about family planning: “*They wanted to know more… like the best methods that they can use for family planning,*” which led to youth facilitators “*teaching them maybe how to use condoms*” or engaging in a ‘family planning educator’ role that they had not been trained for.

The provider focus groups also showed misunderstandings in the purpose and goals of the CSC. While the CSC was intended to allow the community to hold their healthcare clinics and providers accountable for delivering a high quality of care, some providers viewed the CSC as a way to justify or explain poor quality of care and avoid responsibility for quality improvements. When community members expressed frustration at long wait times, providers responded by “telling” and “*mak[ing] them understand that it is a service like any other…so when they come and find a client coming for another service, it is okay for them to wait.*” Providers also used the CSC to justify informal fees to the community as a consequence of stock-outs or a lack of supplies. It appears that some of the providers viewed the CSC as a forum for teaching the community to be more patient or calm. Providers also wanted the community to know that “*if there is anything wrong, they should not escalate it as such because the health care workers are also human beings and they are doing different things so you come and find someone with his or her own…you can never know how they woke up.*” This shows some of the discomfort providers felt with the changing power dynamics brought by the CSC, as they characterized themselves as “trying to moderate” as the CSC facilitators had “*some of that authority [to go] overboard a little bit.*”

While discomfort of some providers with CSC activities emerged slightly in the focus groups, providers were reluctant to clearly criticize the CSC when responding to the short provider questionnaire, with none of the providers characterizing the experience as negative or unsettling. Instead, provider responses during the short provider questionnaire were overwhelmingly positive, such as, “*Love feedbacks both positive and negative…Take negative comments positively in order to change and improve services for clients… Would wish for more activities of score card because they make service provision improve*” (dispensary provider); “*Made providers know weaknesses as well as strengths*” (health center provider); and “*It [the CSC] became an opportunity to be better because gaps were highlighted and as a provider got to work on them. It is a good initiative. Given chance, should be done more often*” (hospital provider).

## Discussion

Recent evidence indicates women in Western Kenya encounter a number of substantial facility-level barriers when seeking family planning, including absent providers, informal payments, and disrespectful treatment (Tumlinson et al*.* 2021a, Tumlinson et al. 2021b, Tumlinson et al. 2021c) [18–20]. Attempts to improve provider performance through training initiatives may not be sufficient to shift provider attitudes and realize quality improvements unless coupled with vastly increased transparency, accountability, and youth-inclusive community participation. We hypothesized larger gains in quality improvement could be achieved through implementation of a youth-led and implemented community score card (CSC). The CSC is designed to facilitate productive patient-provider dialogue, negotiation, and actionable steps for quality improvement. Further, the involvement of young people in the design and implementation of a CSC was hypothesized to give greater voice to this highly vulnerable population.

In reality, quality improvement in the three intervention facilities was inconsistently reported across all modes of evaluation. In the repeated score cards, which represent community *perceptions* of quality improvement, the majority of indicators improved four months after the CSC was introduced – with the largest gains seen in the issues voted highest priority by community members. However, most of the indicators that improved made only small shifts in improvement and—for each facility type—a few indicators stayed stagnant or even worsened. In contrast to the moderate quality improvement seen in the repeated scorecards, data from pre- and post-intervention visits from mystery clients and unannounced enumerators found little to no positive change in provider absenteeism, solicitation of informal payments, disrespectful care, or wait time. While wait time improved at the health center, it worsened at the other two intervention facilities. In many cases—such as the belief that informal payments had substantially improved at the intervention health center—community perceptions were not confirmed with more accurate data collection measures. Importantly, the *perceived* quality improvement shown in the repeated score cards did not translate into increased service delivery. Service statistics revealed a decrease in the percent of women receiving family planning services at intervention facilities before and after the CSC activities, slightly more so than at non-intervention facilities.

Three primary explanations arise to explain the weak performance of the CSC in the context of these three facilities in Kisumu. First, the intervention was implemented during a global pandemic. Additionally, shortly after implementation, clinicians across Kenya went on strike to protest delayed wages and absence of protective gear and hazard pay necessitated by the pandemic. These working conditions likely influenced provider motivation and job satisfaction; an enumerator conducting an unannounced visit reported of one facility: “*Staff speak of lack of pay demotivating them to come to work. Most providers need masks which they say are few.*” The Kenyan ministry of health estimates the pandemic resulted in an approximate ten percent drop in the uptake of essential services [21]- notably the percent of women receiving family planning from public facilities dropped at facilities across the entire sub-county and not solely at our intervention facilities. Expecting dramatic improvements to quality of care within this context may not have been realistic, and even the patchy improvements observed during pandemic conditions may be indicative of the power of community accountability. Secondly, data from our four focus groups indicates large deviations from the intended design of the CSC intervention. Lack of fidelity to the intervention design and purpose likely exacerbated the challenges posed by pandemic conditions in Kisumu. Finally, although we carried out extensive sensitization among providers in participating facilities, it is possible additional efforts are required to ensure providers truly understand the purpose and goals of the CSC—i.e., this tool was not designed for the purposes of teaching healthcare clients to be calmer and more patient—and are able to accept or embrace the resulting shift in the provider–client power dynamic.

While we saw little impact of the CSC on actual quality and service utilization (and only modest improvement in perceived quality), we hypothesize that more rigorous and faithful implementation of the CSC over a longer period of time, coupled with more in-depth community and provider sensitization and a non-pandemic context, could address the larger health system deficiencies experienced by Kenyan women seeking family planning services. It is evident from focus group results that several providers did not follow through on their commitments made during development of the action plan and it is possible that adjustments to implementation could correct for this in future CSC efforts in Kenya.

When implementing the CSC in future community-facility dyads, more attention should be paid to community sensitization so that community members understand that the purpose of the CSC is to improve the quality of family planning service provision, not necessarily to provide education or increase demand for family planning. Additionally, more time and attention should be devoted to facilitator training so that CSC facilitators fully understand their role is to facilitate the score card meetings rather than engage in family planning education. CSC facilitator training should also emphasize that facilitators help guide and clarify the development of the joint action plan, but they are not responsible for carrying out the actions; this is the role of the providers and community members participating in the CSC. Finally, special effort may be needed to help providers embrace the potential for shifting power dynamics within the communities where they work.

One important limitation of this study is that the amount of time between baseline and endline mystery client and unannounced visits was approximately two years. Ideally, the baseline measures would have occurred immediately before intervention implementation. Additionally, the time given for the intervention to take effect was short: just four months as a result of pandemic-related delays to implementation of community gatherings. Ideally, additional monitoring and evaluation of a continuous action plan would occur up to a year following implementation of the initial score card. We also note the small pilot nature of this study limited our capacity to rigorously evaluate the impact of the CSC on the volume of family planning patients served. Finally, the small size of our FGDs allowed for more in-depth conversation of sensitive provider behaviors but may have limited the breadth and range of discussion.

Our findings here dovetail with other recent studies from East Africa that highlight both the promise and complexity of the CSC approach. In 2020, Gullo et al*.* evaluated a CSC intervention in Malawi using a cluster-randomized study design that focused on changes to health workers’ practices and perceptions [22]. Similar to our own results, the authors found several broad areas of improvement but some areas where the CSC did not have its intended impact, including a negative effect on health worker-reported responsibility for HIV testing. A 2020 analysis from Boydell et al*.* similarly underscores the complexity of health worker-community interactions, with a focus on the gendered dynamics and often taboo nature of sexual and reproductive health topics [23]. The authors emphasize the importance of information, dialogue, and negotiation to the success of CSC efforts, finding in their context that the CSC helped to open a forum for frank discussion of family planning and reduce stigma. Our findings add to these results by showing the often-conflicting viewpoints of providers and community members as well as notable differences in perceived versus actual changes to service quality.

There is increased focus among policy makers within the government of Kenya about the rising magnitude of unintended pregnancies among adolescent and young women. In March 2020, the government of Kenya launched a national campaign against teenage pregnancy. Expanding access to family planning services among adolescents and young women is a potentially effective tool in the hands of policy makers for rolling back the burden of unintended pregnancies. Addressing the barriers faced by young unmarried women seeking family planning could go a long way in reducing the large unmet need for family planning in this vulnerable group. The ministry of health has identified adolescents and young women for prioritization in the provision of family planning and is committed to achieving universal access to family planning commodities without discrimination on the basis of age, religion, culture or socio-economic status. However, the quality of family planning services remains suboptimal largely due to interpersonal interactions between providers and their clients, especially in public facilities—which are a major source of family planning commodities for young women [24]. While the ministry of health is committed to delivering family planning services that are client-centered and respect each woman’s human rights [2], there is lack of consensus on how this can be achieved [25]. Ministries of health in LMICs have attempted to improve the quality of family planning services through supportive supervision, technical training, and mentorship [26]. These approaches, however, are ineffective in addressing some of the barriers faced by young women seeking family planning. Social accountability approaches, such as the CSC, could be more effective in addressing interpersonal barriers in family planning provision as this approach empowers direct players (providers and clients) to transform service delivery. Although the impact of the CSC approach in improving the quality of family planning services has not been widely demonstrated and this study had mixed results, other studies have reported reasonable improvement in the quality of other reproductive health services as a result of CSC [22, 27, 28]. Further studies are needed to explore how the CSC approach can be incorporated as a routine intervention in improving family planning services in a cost-effective and sustainable manner.

## Conclusion

Our objectives for this analysis were to assess changes in perceived and actual quality of care as well as changes in the number/percentage of family planning patients served, and to identify barriers and facilitators of the CSC. With respect to perceived quality, the CSC intervention was viewed positively by communities and healthcare providers as a means of collaboratively addressing gaps in the quality of family planning services in three public facilities in Kisumu County, western Kenya. The communities and providers successfully identified barriers to seeking family planning services in public facilities and devised corresponding action plans. Although community members perceived modest improvements in quality, these perceptions were not confirmed by other data methods included in our evaluation and family planning service utilization did not increase post-intervention. Process evaluation identified several barriers to greater intervention success and suggests the need for improved community and provider sensitization prior to CSC implementation;real and lasting quality improvements may have been hampered by Covid-related constraints. More evidence is needed to fully understand the impact of social accountability efforts on contraceptive access.

## Supplementary Information

**Additional file 1.** 

## Data Availability

Mystery client, unannounced visitor, and focus group data are available upon reasonable request to the corresponding author. Kisumu county public-sector service statistics may be obtained from the Kenya Health Information System (KHIS) has been uploaded as a Supplementary file.

## Abbreviations

CHVs: Community Health Volunteers
CSC: Community Score Card
DHIS 2: District Health Information System
FGD: Focus Group Discussion
KEMRI: Kenya Medical Research Institute
KHIS: Kenya Health Information System
LMICs: Low and middle-income countries
YWG: Youth Working Group

## Footnotes

1 At the time of data collection, 200 Kenyan Shillings was equivalent to approximately 1.90 US dollars.

2 There were no Kenyan Ministry of Health sanctioned fees for family planning in any public facilities at the time of data collection.
