# Supplementary material for: Evaluating the feasibility of the Community Score Card and subsequent contraceptive behavior in Kisumu, Kenya
Source: BMC Public Health. 2022 Oct 24;22:1960. doi: 10.1186/s12889-022-14388-y (PMC9592126; doi:10.1186/s12889-022-14388-y)
Supplement: Supplementary file 1 — Additional file 1. [file 12889_2022_14388_MOESM1_ESM.zip › Community action plans.docx]

Appendix: Community Action Plans

**PUBLIC DISPENSARY ACTION PLAN:**

| **NO.** | **IDENTIFIED PROBLEM** | **ACTION ITEMS TO ADDRESS** |
| --- | --- | --- |
| 1 | **Client preferences:** Client preferences for specific family planning methods or providers | -Create awareness in the community of FP services/products and methods  -Sensitize service users to understand capacity of qualified FP providers to offer the services to reduce preferences  -Sensitize parents/young adolescents on FP services  - Lobby for more service providers to address the provider shortage.  - Allow for choices for FP users on providers to improve confidence.  - Build health worker image to address user attitudes |
| 2 | **Illegal user fees:** Health workers charge illegal fees for FP services | -Establish a reporting desk where clients can report demand for payment for FP services |
| 3 | **Confidentiality:** Service providers are not maintaining confidentiality of clients seeking FP, and clients believe that service providers will not maintain confidentiality | -Lobby for the completion of the wing under construction to support increased counseling rooms for FP.  - Create a private room within the facility to offer FP and improve confidentiality for clients  -Sensitize clients during counseling on the need for confidentiality  - Service providers to engage/initiate discussion on FP among clients with teenage children to improve FP discussion with parents  -Support positive parenting skills to improve communication on FP between parents and the youth |
| 4 | **Waiting times, queues, and provider workloads:** how to make sure that FP clients are receiving services outlined in the service charter | -Lobby for improved facilities/recruitment of staff  -Community participation in public participation forums for increased allocation to the facility by the County government  -Service providers to give priority treatment to youths seeking FP services  -Service provides to allocate adequate time while seeking FP services/ change of attitude  -Both parties to respect service times e.g., lunch times.  Scheduling/appointment cards for FP services to be introduced for clients willing to schedule FP services  -The facility to display patient flow chart to guide clients seeking FP services |
| 5 | **Age and gender barriers:** Lack of provision of non-discriminatory FP services, reproductive health services for youth and teens, and lack of community dialogue sessions on FP | -FP services to be offered as per the guidelines and policy.  -Sensitize community members on FP for all  -Complementary health services for male clients such as education, BP, blood sugar etc. to encourage them to attend/ accompany spouses to FP services  -BCC strategies targeting men on FP services |
| 6 | **Stock outs:** All FP commodities are not available year-round | -Improved FP commodity management strategies  - Enhanced use of IT based systems for commodity management  - Lobby for timely procurement and delivery of FP products |
| 7 | Myths and misconceptions: There are myths and incorrect beliefs about FP within the community | -Community sensitization/ awareness  -Improve the facility image within the community  -Improved FP counseling, health information to the community on FP |
| 8 | Fear of HIV testing: FP clients are not being counseled or tested for HIV | -This is a PITC protocol for FP services  -Service providers to explain opt out options to clients during the service |

**PUBLIC HEALTH CENTER ACTION PLAN:**

| **NO.** | **IDENTIFIED PROBLEM** | **ACTION ITEMS TO ADDRESS** |
| --- | --- | --- |
| 1 | **Confidentiality:** Service providers are not maintaining confidentiality of clients seeking FP, and clients believe that service providers will not maintain confidentiality | -Service providers provide assurance to clients before offering the service  -CHVs to provide accurate FP information to the households to avoid self-stigma  -Health facility to develop strategies for parental involvement in FP |
| 2 | **Waiting times, queues, and provider workloads:** how to make sure that FP clients are receiving services outlined in the service charter | -Implement queue management strategies such as numbering  -Front desk staff to explain to the clients/provide information on possible options and time it will take to get FP services  -Implementing patient scheduling techniques.  -The nurse on duty to be housed within the facility |
| 3 | **Age and gender barriers:** Lack of provision of non-discriminatory and non-prejudicial FP services, service providers are not treating all clients equally, and lack of community dialogue sessions on FP | -Support CMEs and CTUs on RH policy and Technology Update.  -Sensitize the community on FP for all.  - Health workers to put in place strategies to reach out to male/partners to support FP |
| 4 | **Client preferences:** Client preferences for specific family planning methods or providers, and do not have adequate information about FP | -Health workers commit to treating all clients equally with respect and dignity  -Health workers to improve the image by practicing customer service  - Health facility to put in place a proper community –facility referral mechanism  -Service providers to respect client’s choices on providers.  -Use CHVs to improve the image of the facility within the community |
| 5 | **Pilfering/Stock outs:** All FP commodities are not available year-round | -Proper planning/forecasting for FP commodities  -Early requisition based on stock levels and reporting of FP commodities  - Enhanced use of IT based systems for commodity management |
| 6 | **Illegal user fees:** FP clients are not able to access services that are free, as per the service charter | -Sensitize all health workers that the FP services are free as per the county government policy  -Establish a reporting/feedback mechanism for reporting demand for bribes |
| 7 | **Illegal abortion services:** teenagers and adolescents have increased access to illegal abortion | - CHVs to identify TBA/Quacks offering abortion services and report to the authorities  -Sensitize the youth on dangers of abortion  -CHVs to conduct follow up for ANC for pregnant youth |

**PUBLIC HOSPITAL ACTION PLAN:**

| **NO.** | **IDENTIFIED PROBLEM** | **ACTION ITEMS TO ADDRESS** |
| --- | --- | --- |
| 1 | **Client preferences:** Client preferences for specific family planning methods or providers, and do not have adequate information about FP | -Create awareness in the community of FP services/products and methods  -Health workers to improve on FP counseling to enable clients to understand FP  -Sensitize parents/young adolescents on FP services  - Community clients to allow adequate time for FP services/counseling |
| 2 | **Illegal user fees:** Health workers charge illegal fees for FP services | -Service providers to explain to the clients that the service is free before offering the service  -Community sensitization on free services and reporting mechanisms  -**Professional** **integrity** for service providers  - Sensitization of clients on Contraceptive method mix to enable client to opt for available methods and avoid being asked to buy products during stock out  -Lobby for supply of commodities e.g., surgical blades for removal of implants |
| 3 | **Confidentiality:** Service providers are not maintaining confidentiality of clients seeking FP, and clients believe that service providers will not maintain confidentiality | -Service providers provide assurance to clients before offering the service  - Providers to strictly observe the code of conduct while providing FP services  - Encourage the youth to use Youth friendly Centre for FP services  -Use disguise methods to conceal services and provide privacy e.g., use of long-sleeved dressing for clients who are on implants  -Have peer FP/Youth FP advocates to facilitate peer discussion in the community.  -Health facility to develop strategies for parental involvement in FP |
| 4 | **Waiting times, queues, and provider workloads:** how to make sure that FP clients are receiving services outlined in the service charter | -implement queue management strategies such as numbering  -Integrate FP services in all service points to increase efficiency  -improve clients' understanding of patient flow at the information desk to reduce waiting time  -Educate the clients on the service process  -Client scheduling e.g., young clients to avoid market days when workload is high |
| 5 | **Age and gender barriers:** Lack of provision of non-discriminatory and non-prejudicial FP services, service providers are not treating all clients equally, and lack of community dialogue sessions on FP | -Support CMEs and CTUs on RH policy and Technology Update  -Sensitize the community on Adolescent Package of care  - Health workers to put in place strategies to target male involvement |
| 6 | **Stock outs:** All FP commodities are not available year-round | -Proper planning/forecasting for FP commodities.  -Early requisition based on stock levels and reporting of FP Commodities.  - Enhanced use of IT based systems for commodity management.  Lobby for timely procurement and delivery of FP products.  -Timely information to clients and community on FP stock information through CHVs and barazas |
| 7 | **Myths and misconceptions:** There are myths and incorrect beliefs about FP within the community | -Community sensitization/ awareness  -Improve the facility image within the community  -Improved FP counseling, health information to the community on FP |
| 8 | **Absenteeism:** Key health workers absent themselves from health facilities | -Health workers schedule/duty roster to be observed |
| 9 | **Rudeness/arrogance of health workers:** Health workers are being rude and arrogant to clients seeking FP services | -Self-awareness for service providers to avoid burn out  -Internal support among health workers in providing FP services  -Treat all clients with respect and dignity.  - Service providers to understand clients' needs and maintain  high standards of professionalism and ethics.  -Community/clients to cooperate in service delivery |
| 10 | **Partner/Male involvement in FP:** There is poor male/partner involvement and support for FP services | -Sensitization of community leaders/male/religious leaders and other community opinion leaders on FP  -Encourage male participation strategies e.g., preferential treatment for couples, services for men |
